# Nanoscale AC Electroosmotic Flow and the Frequency–Size Scaling Observed beyond the Charge Relaxation Regime

**DOI:** 10.1021/acs.nanolett.5c00057

**Published:** 2025-08-08

**Authors:** Gerhard Blankenburg, Huberth Hernández-Alpízar, Leonardo Lesser-Rojas, Chia-Fu Chou

**Affiliations:** † Department of Physics, National Taiwan University, Taipei 10617, Taiwan, R.O.C.; ‡ Nanoscience and Technology Program, Taiwan International Graduate Program, 38017 Academia Sinica, Taipei 11529, Taiwan, R.O.C.; § Institute of Physics, Academia Sinica, Taipei 11529, Taiwan, R.O.C.; ∥ Research Center for Atomic, Nuclear and Molecular Sciences, 27915 Universidad de Costa Rica, San Pedro de Montes de Oca, San José 11501, Costa Rica; ⊥ School of Physics, Universidad de Costa Rica, San Pedro de Montes de Oca, San José 11501, Costa Rica; # Research Center for Applied Sciences, Academia Sinica, Taipei 11529, Taiwan, R.O.C.

**Keywords:** AC electroosmosis (ACEO), nanoscale, electrokinetics, nanofluidics, nanoelectrodes

## Abstract

We present the first observation of AC electroosmosis (ACEO) flow at nanoscale structures in the MHz range, offering new insights into electrokinetic phenomena in this frequency regime. Our findings reveal that ACEO occurs at frequencies much higher than previously anticipated, even surpassing the Debye–Hückel frequency associated with charge relaxation. Despite remaining undetected for over two decades, this result is corroborated by the frequency–size scaling laws derived in this work from the existing ACEO theory. We contextualize these findings within the historical development of electrokinetic phenomena and discuss their implications for the current theoretical framework of ACEO.

## Graphical abstract

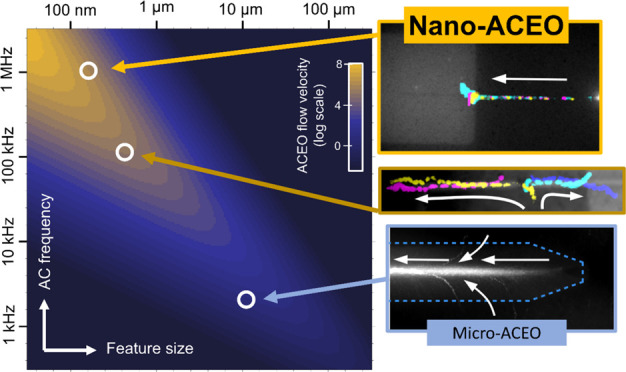

Electrokinetics (EK) in micro- and nanofluidic systems encompass phenomena that employ the unmediated use of electric fields to manipulate particles and fluids.^1,2^ A variety of EK phenomena such as electrophoresis (EP),^3,4^ electroosmosis (EO),^5^ electrothermal flow (ETF),^6^ and dielectrophoresis (DEP)^1,7,8^ have been widely adopted for particles and fluid manipulation. Device miniaturization and implementation of EK with an AC field, such as AC-DEP,^9–15^ further enrich the range of applications. Among all ACEK, AC electroosmosis (ACEO)^16^ and induced-charge electroosmosis (ICEO)^17^ have been known for around two decades. ACEO flow is driven by the periodic charge reversal of the diffuse part of the electric double layer (EDL) which is present over electrode surfaces at the micro- and nanoscale (see Figure 1a). ACEO requires an AC field, since its driving force is generated by the spatial variation of the phase delay of the EDL charging cycle.^18^ ACEO-induced flow is typically directed from edges and corners toward the center of flat electrode structures,^16^ creating characteristic fluid flow rolls.^19^

**Figure 1:**
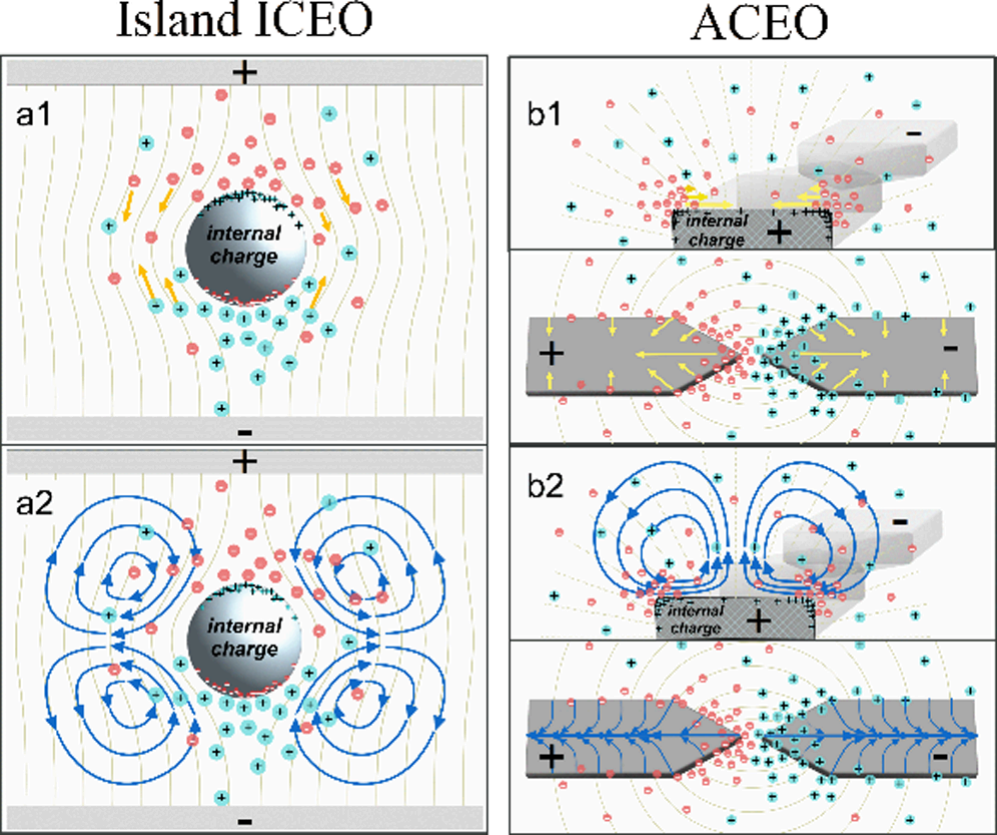
Cartoon sketches of (a1, a2) island ICEO under an external driving field compared to (b1, b2) the ACEO mechanism illustrated by the electrode gap, with schematic depictions of the electric field and accumulated charges. Yellow arrows in (a1, b1) indicate the direction of electrostatic force acting on the ions; dark blue arrows in (a2, b2) indicate the resulting flow patterns.

ICEO has for the most part been identified with another similar yet distinct EO flow, which is generated by the repulsion of the electric field from polarizable island structures (see Figure 1b) and enjoyed significant research activity in recent years.^20^ While based on the same fundamental principles, the mechanisms of island ICEO and ACEO are different (see Figure 1), resulting in contrasting frequency scaling behaviors. This distinction is, however, not well documented in the existing literature. As a consequence, the specific frequency scaling behavior of ACEO has been largely overlooked, with electrokinetic flow in the MHz range often unspecified^21^ or attributed to ETF.^16^

ACEO, ETF and ICEO have been studied extensively on micron-sized structures,^6,22–25^ while the exploration of dielectrophoresis (DEP)^26,27^ has been extended from the microscale to the nanoscale.^28,29^ However, to the best of our knowledge, the transition of the derived scaling laws^30^ to high-frequency ACEO on nanoscaled structures has never been investigated experimentally. As research increasingly focuses on single-molecule DEP experiments,^31–33^ understanding the interaction between targets and electrokinetic phenomena becomes ever more crucial.^27,34,35^ Advancing our knowledge of nanoscale electrokinetics will pave the way for a more precise understanding of biomolecular DEP responses, while also expanding the potential applications of micro- and nanofluidic technologies. In the next section, we briefly introduce the frequency-size scaling relation derived from the conventional ACEO theory, which shall later be compared with our experimental data in the discussion.

In the Gouy–Chapman capacitor model for the EDL^36,37^ in the Debye–Hückel approximation^38^ the EDL thickness is defined by the Debye–Hückel screening length *λ*_*D*_:^38^

1 $$\lambda_{D}=\sqrt{\frac{\varepsilon k_{B}T}{e^{2}\underset{j}{\sum }z_{j}^{2}c_{j,\infty }}}$$

where *ε* is the electric permittivity of the medium, *k*_*B*_ the Boltzmann constant, *T* the absolute temperature and *e* the electron charge, while *z*_*j*_ and *c*_*j,∞*_ are the charge number and the bulk concentration of the ionic species *j*.

The application of the capacitor model to ACEO^18^ requires ionic diffusion to equilibrate across the EDL, hence the common postulate^39^ of the AC frequency to be below the Debye–Hückel frequency *f*_*DH*_, which is given by the reciprocal of the charge relaxation time *τ*_*DH*_:^30^

2 $$\tau_{DH}=\varepsilon /\sigma$$

3 $$f_{DH}≔\frac{1}{2\pi \tau_{DH}}$$

where σ denotes the overall ionic conductivity of the medium.

At higher frequencies, the capacitor model becomes less accurate and other nonlinear effects may interfere with electroosmotic flow,^30,40^ but it is not an imperative limitation for observable EO flow. For more details, please refer to the Supporting Information. From the model, the magnitude of the time-averaged ACEO-inducing stress is found to be

4 $${Τ}_{ACEO}\left(x,f\right)=-4\left(\frac{\Omega^{2}}{{\left(1+\Omega^{2}\right)}^{2}}\right){Τ}_{ACEO}^{\max}\left(x\right)\frac{\nabla \tau_{C}}{\left|\nabla \tau_{C}\right|}$$

where Ω­(*x*, *f*) is a dimensionless frequency variable and *τ*_*C*_ (***x***) is the local charging time of the EDL, and the maximum of the ACEO-inducing stress magnitude at some position ***x***,

5 $${Τ}_{ACEO}^{\max}\left(x\right)\propto \left|\frac{\nabla \tau_{C}}{\tau_{C}}\right|$$

determines the associated frequency scaling of maximized ACEO activity *f*_*ACEO*_^*max*^ (*x*):

6 $$f_{ACEO}^{\max}\left(x\right)\propto \frac{\lambda_{D}}{\tau_{DH}}\left|\frac{\nabla \tau_{C}}{\tau_{C}}\right|$$

where *τ*_*C*_ (***x***) is the local RC charging time of the EDL. This is further expounded in the Supporting Information, while we move on to describe the experiments conducted for this study hereafter.

Downscaling electrode structures from the micro- to the nanoscale comes with certain limitations regarding the achievable sharpness of the fabricated structures. For this study, we have made sure that the width *w*, the height *h* and the minimal radius of curvature *r*_*c*_ in three dimensions of the edges and corners of the electrode structure satisfy the EK standard model postulate of a flat structure with a thin EDL via

7a $$r_{c}<h$$

7b $$r_{c}<\lambda_{D}\ll w$$

Ideally, for the purpose of comparing theory to experiment, one would use the same electrode geometry and material as used in earlier publications^24,30,41^ for which the ACEO theory was derived.^18^ For that reason, we used titanium as an electrode material, since a different electrode material may alter the zeta potential and thus the properties of the observed EO flow.^42^ However, it would be impossible to identify nanoscaled flow patterns on the same electrode geometry as used by those studies by fluorescence microscopy. We circumvented this problem by using two symmetric elongated electrodes with triangular tips pointing at each other (see Figure 2). In this configuration, the optimal condition for the establishment of transversal ACEO flow for varied electrode width *w* was determined. One chip with 15 pairs of micro/nano electrodes (Figure 2a_2_) was bonded to a carrier (Figure 2a_1_) with pins for connection to a voltage source. Microelectrodes of width *w* = 20 μm with internal tip angles of 40° (Figure 2b) were used as a benchmark to link known ACEO scaling behavior^16,18,30^ to the flow patterns observed on the nanoelectrodes.

**Figure 2:**
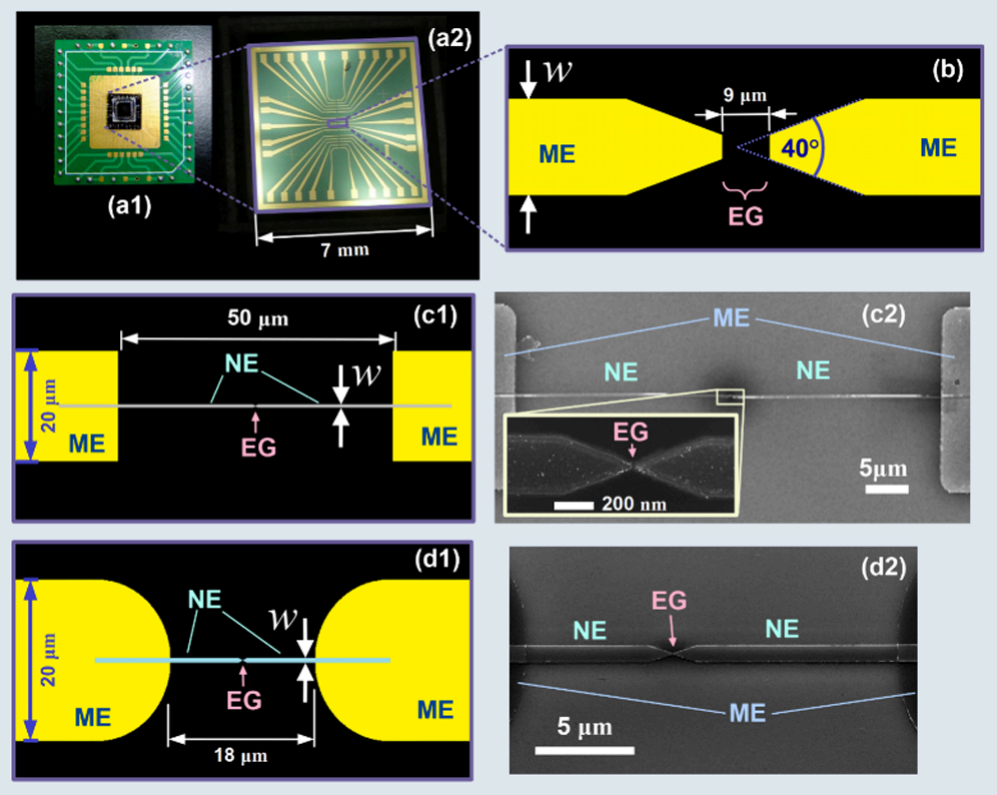
Chip and electrode layout used to study ACEO scaling behavior with electrode size. (a1, a2) Photographs of the device, (b, c1, d1) sketches, and (c2, d2) SEM micrographs of the device. (a1) Assembled chip carrier and (a2) chip with 15 pairs of electrodes. (b) Microsized electrodes *w* = 20 ± 1 μm used for scaling comparison experiments. For experiments with nanoelectrodes, other geometries of the microstructure with larger gaps were chosen in order to reduce their influence on the observed electrokinetic flow. (c1, c2) Design of *w* = 350 ± 20 nm nanoelectrode devices, with the inset showing enlarged environment of the gap area (scale bar 200 nm). (d1, d2) Design used for other devices. The exemplary nanoelectrode shown in (d2) has a width *w* = 960 ± 20 nm. ME: microelectrode, NE: nanoelectrode, EG: electrode gap.

The nanoelectrode device was fabricated by means of UV lithography and e-beam lithography, as described in a previous publication from our group.^29^ For the current experiments, titanium nanoelectrodes of widths 960, 400, and 350 nm were used (Figure 2). All nanoelectrodes had a height of (40 ± 3 nm), radius of curvature *r*_*c*_ ranging from 10 to 20 nm, internal tip angle (60 ± 5°), and gap sizes in the range of 20–30 nm, while only the electrode width *w* was varied between different designs. For the devices with nanoelectrodes (Figure 2c, d), the microelectrodes were fabricated in a different shape, in order to reduce their influence on the observed flow patterns in the relevant frequency ranges.

Carboxylate-modified polystyrene (PS) beads of diameters *d* = 100 nm and *d* = 200 nm (cat. No. F8887, Molecular Probes, Oregon, USA) were suspended in PBS buffer diluted 1:1000 with deionized water (Milli Q) (σ = 30 μS/cm, *λ*_*D*_ ≈ 21 nm). The conductivity was measured with a Horiba conductivity meter (B-173 Twin Cond). Since large amounts of surfactant may alter the dielectric response of the system, the minimal effective concentration of 0.01% M/V Tween20 was added to prevent the beads from sticking to each other or the electrode surface while preserving the observed flow patterns. Subsequently, the fluorescent bead suspension was pipetted onto the center of the chip and enclosed by a ring of double-sided tape and a small circular coverslip.

The probe was observed with a Leica microscope (DMI 4000 B) using a B/G/R filter (for more details see Supporting Information) and a 100x oil-immersion objective. Kinetic series were recorded with an Andor camera (IXON DV887), at 40 fps and 4.3 ms exposure for *d* = 100 nm beads, and at 100 fps and 8.75 ms exposure (using frame transfer) for *d* = 200 nm beads. AC voltage was generated with a function generator (Agilent 33220A). Simulations were conducted using COMSOL version 5.5 for a water volume of 3 μm (*x*) by 1 μm (*y*) by 1 μm (*z*) above the substrate centered around the electrode gap. The electrodes in the simulation were 400 nm wide with a 20 nm gap, and with rounded edges and corners with a radius of curvature of *r*_*c*_ = 10 nm (Figure 3a-b).

**Figure 3:**
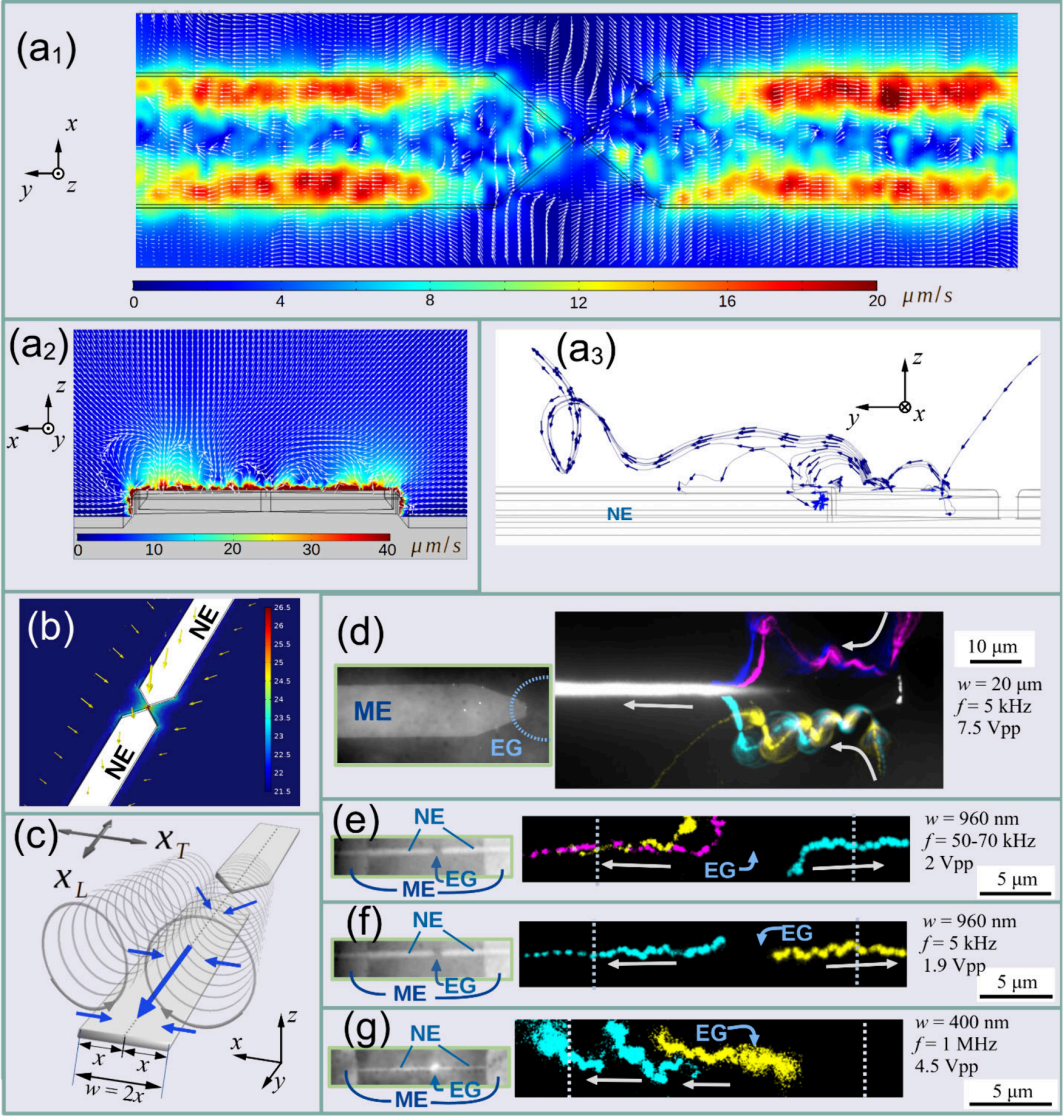
Evidence of nanoscale ACEO from simulation and experiment. (a_1_–a_3_) COMSOL simulation of ACEO-induced flow over a nanoelectrode. (a_1_) Top view (flow at 10 nm above the electrode surface), (a_2_) selected cross-section at 500 nm distance from the nanogap in the *y* direction, and (a_3_) stream lines of the calculated flow field in the vicinity of the nanoelectrodes. The electrode width in the simulation was 400 nm, and the electrode gap size was 20 nm. Electrodes were modeled with a radius of curvature *r*_*c*_ = 10 nm for more realistic results. Supplementary Video S1 is an animation of successive cross-sectional slices along the *y*-axis starting from the nanogap (*y* = 0 nm) up to a distance of 1.5 μm from the gap. (b) Direction and logarithmic magnitude of the gradient of the squared electric field near the same COMSOL model electrode. (c) Schematic cartoon of the flow rolls observed over the electrodes in the experiment. Blue arrows indicate the flow direction. Crossed gray arrows indicate the longitudinal (*x*_*L*_) and transversal (*x*_*T*_) direction with respect to the electrodes. The flow pattern is only depicted over the left electrode for better visibility. The variable *x* used in the equations corresponds to half the electrode width. Cartesian principal directions *x, y*, and *z* are defined for comparison with (a_1_–a_3_). (d–g) Spiraling traces of fluorescent beads observed over electrodes of various sizes (see corresponding Videos S2 and S3 in the Supporting Information). Arrows indicate the direction of movement. Traces were produced by projecting 100 frames of an image stack recorded at 100 fps; images were recorded using the frame-transfer mode, exposure time 8.75 ms, using *d* = 200 nm fluorescent PS beads. Yellow, magenta, cyan, and blue markings represent traces of different individual particles recorded at different times, which were composed together into one image.

In the experiment, a distinctive flow pattern formed over each pair of electrodes in a characteristic frequency range depending on the electrode size (Figure 3c-g). Along the edges of each electrode, two parallel cylindrical fluid rolls were observed, as illustrated in a schematic sketch in Figure 3c. Projected microscope images in Figure 3d show detailed evidence of this flow pattern on the microelectrodes. The observed flow rolls were symmetric on both electrodes and rotated in the transversal (*x*_*T*_) direction with respect to the main direction of the electric field (Figure 3c) moving the fluorescent beads toward the center of the electrodes. From the center of the electrodes, the beads continued in a conveyor-belt-like motion along the electrode axis.

Similar flow patterns were reproduced on smaller-scaled electrodes at higher frequencies by experiment (Figure 3e-g) and simulation (Figure 3a_1_-a_3_) following the approach of Yoshida et al.^43^ In the experiment, evidence of flow rolls was observed in the form of sporadic spiraling bead movements on the larger-scaled electrodes (Figure 3d-f). On electrodes below a certain width (*w* ≤ 400 nm, Figure 3g), no sufficient evidence for these spiral patterns was seen. Such flow patterns may be too small to be resolved under the microscope or the used fluorescent beads may also be too large to follow such small flow patterns. However, the conveyor-belt motion was still observable, exhibiting the characteristic frequency scaling of ACEO flow with electrode size. Interestingly, the simulation results indicate a more complex structure of the flow field on the nanoscaled electrodes both in the substrate *x*–*y* plane 10 nm above the electrode surface (Figure 3a_1_) as well as the cross-sectional *y*–*z* plane at 500 nm distance from the nanogap (Figure 3a_2_), as compared to larger-scaled electrodes. Figure 3a_3_ shows simulated stream lines of ACEO flow over the surface of an electrode of width *w* = 400 nm, confirming the ACEO origin of the above-mentioned conveyor-belt flow pattern. Since the used fluorescent beads were of diameter *d* ≥ 100 *nm*, they were likely not affected by the smaller-scale patterns of changing flow direction indicated in Figure 3a_1_ and 3a_2_, but rather followed the larger-scale collective flow at some distance above the electrode, which is depicted in Figure 3a_3_.

Figure 4 shows the flow patterns observed at varied frequencies over the microelectrodes of width *w* = 20 μm, which were used to reproduce literature results,^16,18,30^ compared to nanoelectrodes of widths 960 and 350 nm. On the microelectrodes (Figure 4a), the development of the flow pattern into a single straight line centered over the electrode axis could be observed as the frequency was tuned to the optimal ACEO range at several kHz. This is in perfect agreement with the well-known regime of ACEO phenomena.^16^ An equivalent pattern was observed on the nanoelectrodes at significantly higher frequencies, in accordance with the expected scaling behavior of ACEO (eq 6).^30^ The pattern on the *w* = 960 nm electrodes (Figure 4b) was well-defined around the frequency of 100 kHz, suggesting that this condition generates the most stable fluid rolls along the electrode edges. At frequencies above 250 kHz, the flow pattern gradually dispersed away from the longitudinal axis at larger distances from the nanogap, until a pattern with radial symmetry centered around the electrode nanogap emerged at about 500 kHz. At frequencies below 100 kHz, the pattern became more disperse near the nanogap while the waist of the pattern moves away from the gap toward the microelectrodes. Larger beads (*d* = 200 nm) were used for these electrodes since they are more clearly visible and can be observed at higher velocities.

**Figure 4:**
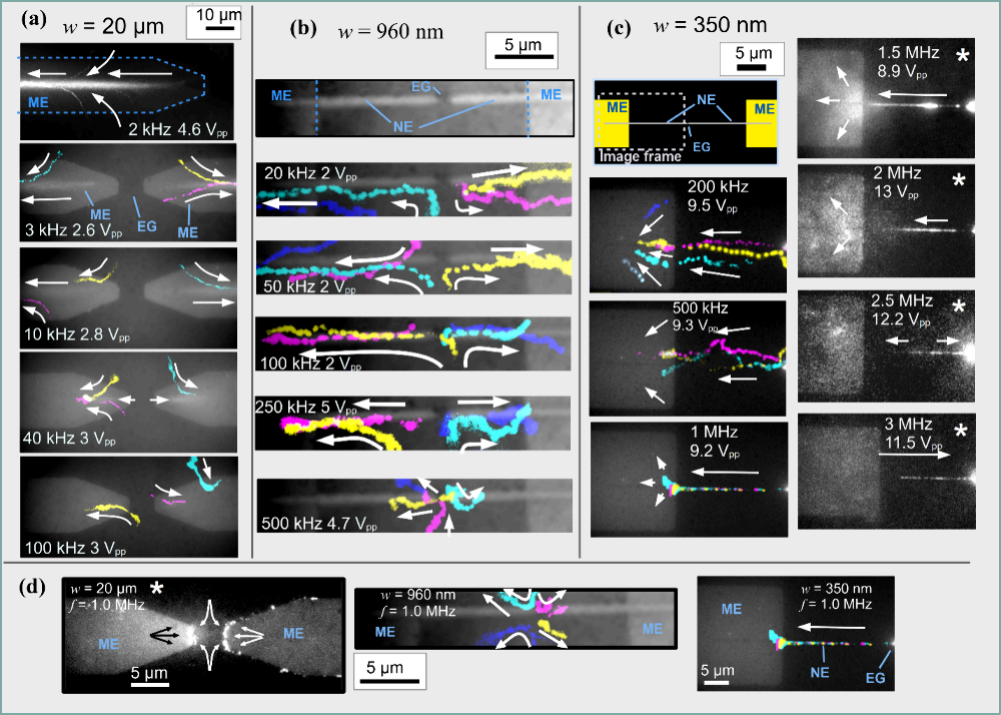
Transition to high-frequency ACEO flow from microelectrodes to nanoelectrodes. (a–c) Observed ACEO frequency scaling with electrode width, showing a shift of ACEO activity to higher frequencies as the electrode size is downscaled. Exemplary observed particle velocities ranged from O­(1 μm/s) to O­(100 μm/s), as reported in Table S1 in the Supporting Information. (a) *w* = 20 μm microelectrodes (see Supplementary Video S4), (b) *w* = 960 nm nanoelectrodes (see Supplementary Video S5), (c) *w* = 350 nm nanoelectrodes (see Supplementary Video S6); schematic pictures above show the electrode location. (d) Comparison of the observed flow patterns at *f* = 1 MHz on electrodes of different widths (see Supplementary Video S7). Arrows indicate the direction of particle motion. Yellow, magenta, cyan, and blue markings represent traces of different individual particles recorded at different times, which were composed together into one image. Grayscale images with an asterisk represent conditions with particle accumulation rather than translation, where individual particle traces cannot be reconstructed, showing an image of the time-averaged distribution instead. Images in (a) were recorded at a frame rate of 40 fps, using *d* = 200 nm fluorescent PS beads, in (b) at a frame rate of 100 fps, with the same beads, and in (c) at a frame rate of 40 fps, using *d* = 100 nm beads.

The transition to MHz ACEO is shown in Figure 4c on nanoelectrodes of width *w* = 350 nm. In this case smaller beads (*d* = 100 nm) were used, since they are more likely to get caught in smaller-scaled flow rolls and have a more favorable ratio of ACEO-induced force compared to DEP force.^44^ The pattern was again observed at higher frequencies compared to the *w* = 960 nm electrodes. At frequencies up to 500 kHz, the fluorescent beads were not collected over the electrode axis, despite positive DEP force as indicated by a high accumulation in the nanogap. The beads were collected over the electrode axis in the range from 1 MHz up to 3 MHz, far beyond the Debye–Hückel frequency (*f*_*DH*_ ≈ 690 kHz), as stated by eq 3. The longitudinal, conveyor-belt movement along the nanowire underwent direction reversal in the range between 2 and 3 MHz. However, the beads were located over the electrode axis throughout the displayed frequency range. This indicates that the transversal component of the cylindrical flow maintained its direction, while the longitudinal component was reversed in direction. Notably, the three electrodes of different widths exhibited significantly different flow patterns at 1 MHz AC frequency, as shown in Figure 4d. All of these observations strongly suggest that the transversal flow rolls are caused by ACEO, as will be further elaborated in the next section.

***ACEO-induced flow*** exerts force on the ion distribution of the EDL and can be easily confused with other electrokinetic phenomena due to their similar flow patterns,^30^ namely electrothermal flow (ETF), which acts on the bulk medium, and DEP, which arises from the dielectric interplay between the medium and the target in nonuniform electric fields. Nevertheless they can be distinguished from ACEO because of subtle differences in the pattern geometry and characteristic frequency scaling,^16,44^ which in contrast to ACEO is independent of the electrode size. In the following section, we argue why ACEO can be identified as the main cause of the observed flow patterns and demonstrate the agreement of our observations with the well-established theory.

***ETF*** undergoes a change in flow direction at a characteristic frequency^16^ around *f* = 3*f*_*DH*_ (see Supporting Information for more details). Near the tips, a change in flow direction was observed for *w* = 350 nm electrodes in that frequency range, indicating for a contribution of ETF.

Positive ***dielectrophoretic (DEP)*** force may act superimposed on ACEO, aiding in the accumulation of beads (Figure 3b). Conversely, *negative DEP* of the larger beads appeared to hinder bead accumulation at higher frequencies. On the microelectrodes, DEP-like accumulation can still be observed at 1 MHz, in agreement with the literature.^30^ In contrast, the particle motion at the same frequency appeared to be dominated by ETF on the 960 nm wide electrodes and by ACEO on the *w* = 350 nm electrodes with smaller beads (see Figure 4d).

The crossover frequency of the DEP force^44^ scales with the particle size, but not with the electrode size, and the DEP force is strongest near the electrode nanogap, as shown by our simulation (Figure 3b). By contrast, movement or collection of the same (*d* = 200 nm) type of PS beads along the electrodes, away from the nanogap, was observed in a much higher frequency range for *w* = 960 nm electrodes than for the microelectrodes, as shown by the projections in Figure 4a-b. This trend continued toward electrodes of width *w* = 400 nm, as shown in the Supplementary Video S8. However, in that experiment the observation of ACEO at frequencies above 800 kHz was hindered by *negative DEP force* repelling those beads from the electrodes. Notably, the observed sustained longitudinal movement of the beads above the electrodes and subsequent transport from the nanoelectrodes onto the surface of the microelectrodes (see Figure 4) is expected with transversal flow rolls like the ones depicted in Figure 3 and ACEO, but not with DEP.

In the following order-of-magnitude analysis, we demonstrate that the characteristic relationship of size scaling and frequency scaling corresponds to the expectation for ACEO-induced flow based on standard literature.^19^ The scaling laws^30^ were derived for a specific electrode geometry where, due to symmetry, there is no transversal flow and longitudinal ACEO can be approximated in two dimensions. While an exact theory for both transversal and longitudinal ACEO scaling on our (three-dimensional) electrodes will have to be elaborated in the future for a quantitative comparison, eqs 4–6 hold for any electrode geometry and flow direction. Rough numbers of the observed longitudinal particle, ranging up to several hundred *μm*/*s*, are presented in Table S1 of the Supporting Information. While these numbers are in general agreement with the simulated velocities (up to 20 *μm*/*s*), presented in Figure 4a, this movement may be influenced by other EK phenomena in a manner yet to be unraveled, as exemplified by the apparent reversal in flow direction between 2 and 3 MHz. A simplified theoretical draft, which is presented in the Supporting Information, suggests a similar behavior for the transversal effect on our electrodes as reported for the longitudinal effect in the literature. For a qualitative estimate of the scaling behavior, it is therefore justifiable to identify the parameter *x* from that theory with the half electrode width (*w/2*) in our experiment. Based on the model,^18^ the scaling of the ACEO-induced speed can be calculated for varied parameters *f* and *x* using eq 4, as plotted in Figure 5 (the calculations are demonstrated in the Supporting Information). It can be seen in Figure 5b and 5c that the maximum is shifted toward higher frequencies as the size parameter *x* is lowered and vice versa. This is in perfect agreement with the analysis of our experimental results, which are represented by black circles in the speed contour map in Figure 5a. Close inspection of the model derivation^18^ reveals that the reciprocal of the charge relaxation time is not an imperative constriction to the phenomenon as such, but rather a limit for the validity of the EDL model^38^ and analytical derivations built thereon. Note that the graph shows the expected flow speed and ignores edge effects; the threshold to maintain a certain flow pattern may be different at different size scales of the flow rolls, since competing phenomena such as Brownian motion interact differently at different scales.

**Figure 5:**
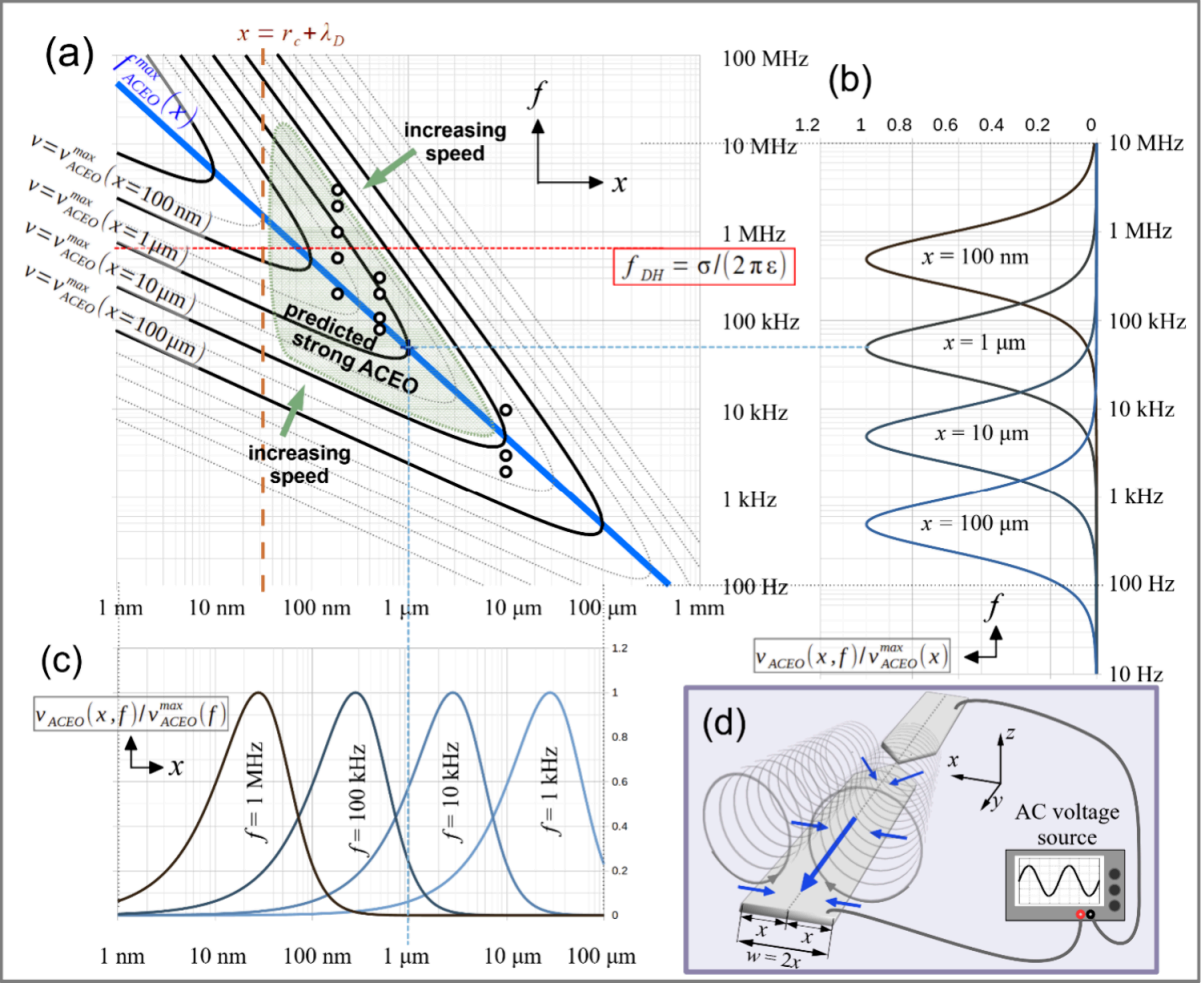
ACEO-induced speed scaling versus frequency and distance at a fixed voltage. (a) Speed contour map (black and gray solid lines are iso-speed curves) in an *f*–*x* phase diagram. Each black curve corresponds to a speed value equal to the maximum at a specific size scale (see labels on the left). The blue line marks the points *f*_*ACEO*_^*max*^(*x*) of maximized ACEO speed at fixed *x*, as calculated via eq 6. The solid red line indicates the value of the Debye–Hückel frequency. An orange dashed line marks the sum of the radius of curvature (*r*_*C*_) and the Debye length (*λ*_*D*_) in the experiments. Black circles indicate the parameters of the observations presented in this work. (b) Normalized ACEO-induced speed *v*_*ACEO*_(*x, f*)/*v*_*ACEO*_^*max*^(*x*) vs frequency (*f*). (c) Normalized ACEO-induced speed *v*_*ACEO*_(*x, f*)/*v*_*ACEO*_^*max*^(*f*) vs length scale (*x*). (d) Schematic of the system indicating the scaling parameter *x*.

To our knowledge, this study presents the first report of ACEO flow at nanoscale structures in the MHz range, marking a potentially significant advancement in understanding electrokinetic phenomena within this regime. We demonstrated that ACEO occurs at frequencies far higher than previously anticipated. Although overlooked for over two decades, this result was implicitly predicted by existing ACEO theory. We discussed these findings in the context of the historical progression of electrokinetic phenomena and their implications for the current theoretical model of ACEO. Our findings reveal that on the nanoscale, ACEO activity scales toward MHz frequencies as electrode structures are reduced in size and that ACEO flow persists even beyond the Debye–Hückel frequency associated with charge relaxation, challenging conventional understanding. These results underscore the need for more detailed investigations into nanoscale electrokinetics to enhance our ability to distinguish and predict the phenomena influencing nanofluidic experiments. With growing interest in low-copy-number biomolecular manipulation and sensing using AC field, such as DEP-based experiments,^21,27–29,35,45–47^ further research is needed in numerical simulation, experimental investigation, and analytical theory of nanoscale electrokinetic phenomena.

## Supplementary Material



















## Notes

Experimental details; electrode geometry in ACEO theory; mathematical derivation for Figure 5; ETF theory elaboration; list of supplementary videos with detailed descriptions (PDF)
Video S1: Fly-by animation of simulated ACEO flow (MP4)
Video S2: Flow spirals on microelectrodes (MP4)
Video S3: Flow patterns on *w* = 960 nm electrodes up to 100 kHz (MP4)
Video S4: Flow patterns on *w* = 20 μm electrodes (MP4)
Video S5: Flow patterns on *w* = 960 nm electrodes up to 1 MHz (MP4)
Video S6: Flow patterns on *w* = 350 nm electrodes (MP4)
Video S7: Flow patterns at 1 MHz on various electrodes (MP4)
Video S8: Flow patterns on *w* = 400 nm electrodes (MP4)
