# Supplementary material for: Nanoscale AC Electroosmotic Flow and the Frequency–Size Scaling Observed beyond the Charge Relaxation Regime
Source: Nano Lett. 2025 Aug 8;25(35):13128–35. doi: 10.1021/acs.nanolett.5c00057 (PMC12412179; doi:10.1021/acs.nanolett.5c00057)
Supplement: Supplementary file 1 [file nl5c00057_si_001.pdf]

Supplementary Materials

Nanoscale AC Electroosmotic Flow and the Frequency-Size Scaling Observed Beyond the Charge Relaxation Regime

*Gerhard Blankenburg[1,2,3], Huberth Hernández-Alpízar[4], Leonardo Lesser-Rojas[4,5], Chia-Fu Chou[3,6,]**

[1] Department of Physics, National Taiwan University, Taipei 10617, Taiwan, R.O.C.

[2] Nanoscience and Technology Program, Taiwan International Graduate Program, Academia Sinica, Taipei 11529, Taiwan, R.O.C.

[3] Institute of Physics, Academia Sinica, Taipei 11529, Taiwan, R.O.C.

[4] Research Center for Atomic, Nuclear and Molecular Sciences, Universidad de Costa Rica, San Pedro de Montes de Oca, San José 11501, Costa Rica

[5] School of Physics, Universidad de Costa Rica, San Pedro de Montes de Oca, San José 11501, Costa Rica

[6] Research Center for Applied Sciences, Academia Sinica, Taipei 11529, Taiwan, R.O.C.

*Correspondence: cfchou@phys.sinica.edu.tw

Experimental details

Optical filter

B/G/R (Leica Fluorescence)

Excitation filter: BP420/30 BP 495/15 BP 570/20

dichroic mirror: 415 510 590

Suppression filter: 465/20 530/30 640/40

LED details

MIGHTEX LSC-0560-03-XX

central wavelength 560 nm (FWHM roughly 100 nm)

nominal output power 240 mW

Fluorescent beads

Molecular Probes FluoSpheres Red fluorescent (580/605) size kit #1 (catalogue No. F8887) carboxylate-modified microspheres, the used diameters were 0.1 μm and 0.2 μm.

Excitation range: roughly 480 ~ 580 nm

Emission range: roughly 580 ~ 680 nm

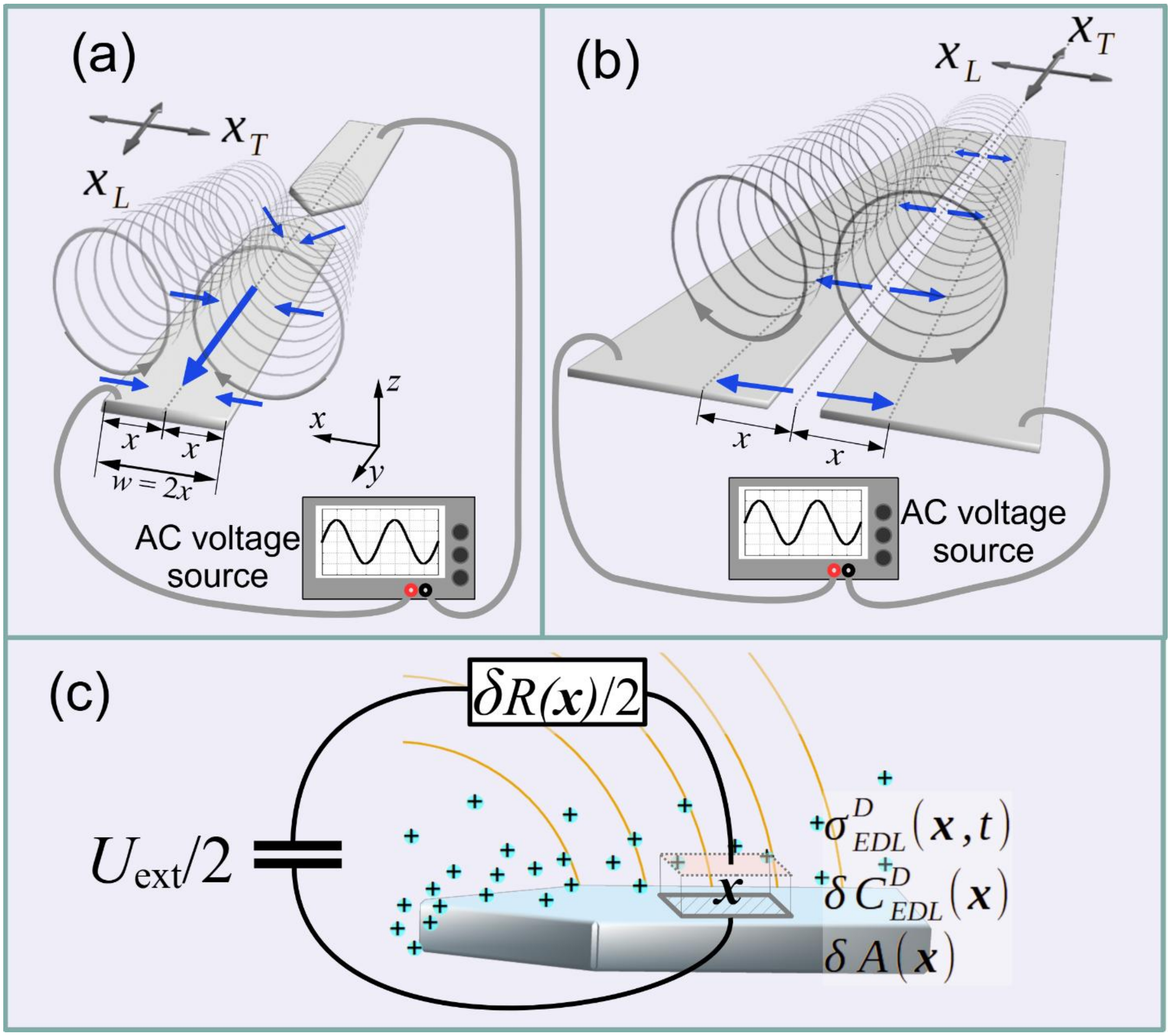

Figure S1. A direct comparison of the basic geometry of our device (a) with the 'parallel finger' electrode geometry described in earlier publications[1] and (b) on which the ACEO model[2, 3] is based. The relevant size coordinate x as well as the transversal (x_T) and longitudinal (x_L) directions with respect to the electrodes are marked for both cases. (c) Schematic sketch for the derivation of the ACEO-induced velocity. For a given point x on the electrode surface, an infinitesimal surface element centered around x is chosen, with an area $\delta A(x)$ and an associated diffuse EDL with an infinitesimal capacitance $\delta C^D_{EDL}(x)$ and interfacial charge density $\sigma^D_{EDL}(x,t)$.

Observed flow velocities

Table S1 shows a list of the rough range of some exemplary particle velocities observed in the videos represented by Fig. 4a-c of the main text. These represent a few random samples of particles that happened to be trackable for each video and in no way constitute a complete or statistically accurate set of measured velocities which would enable us to infer quantitative conclusions. All of these were measured as longitudinal particle speeds, where positive and negative values indicate a flow direction away from and towards the nanogap, respectively.

Table S1. Observed longitudinal flow speeds in the videos presented in Fig. 4 of the main text.

w	$x = w/2$	f	U	$observed\ speed\ range$
350 nm	175 nm	200 kHz	9.5 V_{pp}	$30 \cdots 60\ \mu m/s$
350 nm	175 nm	500 kHz	9.3 V_{pp}	$100 \cdots 106\ \mu m/s$
350 nm	175 nm	1.0 MHz	9.2 V_{pp}	$40 \cdots 110\ \mu m/s$
350 nm	175 nm	1.5 MHz	8.9 V_{pp}	$50 \cdots 110\ \mu m/s$
350 nm	175 nm	2.0 MHz	13.0 V_{pp}	$40 \cdots 100\ \mu m/s$
350 nm	175 nm	2.5 MHz	12.2 V_{pp}	$30 \cdots 60\ \mu m/s$
350 nm	175 nm	3.0 MHz	11.5 V_{pp}	$-40 \cdots -10\ \mu m/s$
960 nm	480 nm	20 kHz	2.0 V_{pp}	$20 \cdots 36\ \mu m/s$
960 nm	480 nm	50 kHz	2.0 V_{pp}	$30 \cdots 80\ \mu m/s$
960 nm	480 nm	100 kHz	2.0 V_{pp}	$100 \cdots 140\ \mu m/s$
960 nm	480 nm	250 kHz	5.0 V_{pp}	$200 \cdots 400\ \mu m/s$
960 nm	480 nm	500 kHz	4.7 V_{pp}	-
20 µm	10 µm	2 kHz	2.3 V_{pp}	$20 \cdots 36\ \mu m/s$
20 µm	10 µm	3 kHz	2.6 V_{pp}	$30 \cdots 80\ \mu m/s$
20 µm	10 µm	10 kHz	2.8 V_{pp}	$20 \cdots 46\ \mu m/s$
20 µm	10 µm	40 kHz	3.0 V_{pp}	-
20 µm	10 µm	100 kHz	3.0 V_{pp}	-

The basic picture of ACEO

In ACEO, flow is generated mostly near edges and corners of a polarizable surface, exclusively in AC fields, by a **dynamic phase delay** of the EDL charging cycle[1] when the electric field is expelled from the medium. The ions present in the incompletely charged EDL are driven to equilibrate the EDL saturation level across the electrode surface, generating ACEO flow. In this analysis, we follow the approach by Castellanos *et al.*[2], focusing on qualitative scaling behavior rather than quantitative computation of the ACEO velocity.

Consider an infinitesimally small surface section of area $\delta A(\boldsymbol{x})$ centered around a point $\boldsymbol{x}$ on the electrode surface with an associated EDL surface charge density $\sigma_{EDL}^{D}(\boldsymbol{x},t)$ and local diffuse EDL capacitance $\delta C_{EDL}^{D}(\boldsymbol{x})$. The corresponding segment of the diffuse EDL is charged via the integrated resistivity $\delta R(\boldsymbol{x})$ of the tube-like slice of electrolyte formed by the field lines passing through the surface section as they extend through space towards the opposite electrode (see Fig. S1c). Introducing the viscosity of the medium η, the known expression[1] for the time-averaged ACEO-induced velocity at the point $\boldsymbol{x}$ on a flat electrode surface is:

$$\boldsymbol{v}_{ACEO}(\boldsymbol{x}) = \frac{\lambda_D}{\eta}\left\langle \Re\left(\boldsymbol{T}_{ACEO}(\boldsymbol{x},t)\right)\right\rangle_t \qquad \text{(S1)}$$

with the ACEO-inducing stress given by

$$\boldsymbol{T}_{ACEO}(\boldsymbol{x},t) = \sigma_{EDL}^{D}(\boldsymbol{x},t)\boldsymbol{\nabla}U_{EDL}^{*}(\boldsymbol{x},t), \qquad \text{(S2)}$$

where the gradient of the potential drop across the EDL (the zeta potential), $U_{EDL}(\boldsymbol{x},t)$, is used to approximate the component of the electric field parallel to the electrode surface experienced by the ions in the EDL. The velocity of the observable flow pattern depends on how the ACEO-inducing stress of different parts of the electrode surface work in concert, hence in a general case it will differ from the expression in Eq. S1. However, the expression for the local ACEO-inducing stress as stated in Eq. S2 remains valid for any electrode geometry.

The role of charge relaxation in ACEO

This section is meant as a supplement to the paragraph about "Time scales for ICEO flows" by Squires et al.[3], highlighting unique aspects of ACEO that were not discussed in that work.

Two fundamental processes are involved in the generation of ACEO flow: on the one hand the charge relaxation time τ_{DH}, as stated in Eq. 2 of the main text, on the other hand the charging time of an equivalent RC circuit[1, 2], $\tau_C(\boldsymbol{x})$. While τ_{DH}, which is based on the Debye-Hückel picture, is an intrinsic property of the ionic solution accounting for ionic diffusion across the thickness of the EDL, $\tau_C(\boldsymbol{x})$ scales with the electrode size because, in the general case of an arbitrary electrode geometry, δC_{EDL}^D and δR are dependent on $\boldsymbol{x}$:

$$\tau_C(\boldsymbol{x}) \propto \delta C_{EDL}^D(\boldsymbol{x}) \cdot \delta R(\boldsymbol{x})/2. \tag{S3}$$

Since ACEO only occurs in AC fields, a time-dependent sinusoidal AC potential oscillating at the frequency f is used in the following discussion:

$$U_{ext}(t) = U_0 e^{2\pi i f t}\ , \tag{S4}$$

where i is the imaginary unit. The equivalent circuit model[1] can then be employed to find:

$$\sigma_{EDL}^D(\boldsymbol{x},t) = \frac{\delta C_{EDL}^D(x)}{\delta A(x)} U_{EDL}(\boldsymbol{x},t) \tag{S5}$$

$$U_{EDL}(\boldsymbol{x},t) = \frac{U_{ext}(t)}{2\left(1+2\pi i f \tau_C(x)\right)} \equiv \frac{U_{ext}(t)}{2(1+i\Omega)} \tag{S6}$$

with the dimensionless frequency variable $\Omega \equiv \Omega(\boldsymbol{x},f) \coloneqq 2\pi f \tau_C(\boldsymbol{x})$, as stated in the main text.

The effect of the charge transport is a delayed accumulation of charges in the equivalent capacitor of the EDL. The time-dependent EDL charge density is

$$\sigma_{EDL}^D(\boldsymbol{x},t) = \Re\left(\frac{\delta C_{EDL}^D}{\delta A} \frac{U_{ext}(t)}{2(1+i\Omega)}\right) = \frac{\delta C_{EDL}^D}{\delta A} \frac{U_0}{2\sqrt{1+\Omega^2}} \left(\cos(\Omega(t/\tau_C) + \tan^{-1}(\Omega))\right) \tag{S7}$$

Electrode geometry in ACEO theory

ACEO theory[1, 2] was first derived for a specific electrode geometry, termed 'parallel finger electrodes' (see Fig. S1b), which differs from the geometry used in this work (see Fig. S1a). In this section, the role of electrode geometry in ACEO is briefly discussed based on the derivation of ACEO velocity by Ramos et al.[1]. Starting from Eq. S6, the local gradient of the zeta potential can be derived:

$$\boldsymbol{\nabla} U_{EDL}^{*}(\boldsymbol{x}, t) = \left(\frac{-i\omega U_{ext}^{*}}{2(1+i\omega\tau_C)^2}\right)\left(\nabla\tau_C(\boldsymbol{x})\right) = \left(\frac{-i\Omega U_{ext}^{*}}{2(1+i\Omega)^2}\right)\frac{\boldsymbol{\nabla}\tau_C}{\tau_C} \quad \text{(S8)}$$

Plugging this and Eq. S7 into Eq. S2, one obtains

$$\boldsymbol{T}_{ACEO} = -\frac{\delta C_{EDL}^{D}}{\delta A}\frac{U_0^2}{8}\left(\frac{\Omega^2}{(1+\Omega^2)^2}\right)\frac{\boldsymbol{\nabla}\tau_C}{\tau_C} \quad \text{(S9)}$$

The expression $\boldsymbol{\nabla}\tau_C/\tau_C$ is independent of the AC frequency (see Eq. S3). Therefore, the direction of the local stress will not change when the frequency is varied, and the maximum of the ACEO-inducing stress magnitude at a given position $\boldsymbol{x}$ on the electrode surface, $T_{ACEO}^{max}(\boldsymbol{x})$ and corresponding AC frequency $f_{ACEO}^{max}(\boldsymbol{x})$ are obtained by maximizing the expression in brackets $\left(\frac{\Omega^2}{(1+\Omega^2)^2}\right)$, which is achieved by setting $\Omega = 1$:

$$f_{ACEO}^{max}(\boldsymbol{x}) = (2\pi\tau_C)^{-1} \quad \text{(S10)}$$

$$T_{ACEO}^{max}(\boldsymbol{x}) = \frac{\delta C_{EDL}^{D}}{\delta A}\frac{U_0^2}{32}\left|\frac{\boldsymbol{\nabla}\tau_C}{\tau_C}\right| \quad \text{(S11)}$$

In most cases, especially for flat, homogeneous surfaces, $\frac{\delta C_{EDL}^{D}(x)}{\delta A(x)}$ is constant (to the first order) and in order to solve ACEO scaling, it suffices to determine the local RC charging time of the EDL, $\tau_C(\boldsymbol{x})$.

For example, in the case of the 'parallel finger electrodes', where x denotes the the distance from the electrode gap, as depicted in Fig. S1b, with a cylindrical electric field employing the

approximation for a flat EDL:

$$\frac{\delta C_{EDL}^{D}}{\delta A} = \frac{\Lambda\varepsilon}{\lambda_D}, \tag{S12}$$

one finds:

$$\delta R \delta A = \frac{\pi x}{\sigma} = \frac{\pi x \tau_{DH}}{\varepsilon};$$

$$\tau_C = \frac{\delta R * \delta C_{EDL}^{D}}{2} = \frac{\delta C_{EDL}^{D}}{\delta A}\frac{\pi \tau_{DH}}{2\varepsilon} x = \frac{\Lambda \pi \tau_{DH}}{2\lambda_D} x$$

$$\left|\frac{\nabla \tau_C}{\tau_C}\right| = x^{-1},$$

$$f_{ACEO}^{max}(x) = \frac{\delta A}{\delta C_{EDL}^{D}}\frac{\varepsilon}{\pi^2 \tau_{DH}} x^{-1} = \frac{\lambda_D}{\Lambda \pi^2 \tau_{DH}} x^{-1} \tag{S13}$$

$$\boldsymbol{T}_{ACEO}(x, f) = -\left(\frac{\delta C_{EDL}^{D}}{\delta A}\frac{U_0^2}{8}\left(\frac{\Omega^2}{(1+\Omega^2)^2}\right)x^{-1}\right)\hat{\boldsymbol{x}} = -\left(\frac{\Lambda\varepsilon}{\lambda_D}\frac{U_0^2}{8}\left(\frac{\Omega^2}{(1+\Omega^2)^2}\right)x^{-1}\right)\hat{\boldsymbol{x}}, \tag{S14}$$

$$T_{ACEO}^{max}(x) = \left(\frac{\delta C_{EDL}^{D}}{\delta A}\right)\frac{U_0^2}{32} x^{-1} \tag{S15}$$

reproducing the result presented by Ramos et al., as presented in Eq. 4 of the main text.

In a more general scenario, like the case of transversal ACEO, and especially on the nanoscale, the effect of curved surfaces may lead to variations in the result caused, among others, by deviating EDL capacitances and more appreciable variations of the RC charging characteristics over small distances. In such cases, quantitative predictions of the observable flow pattern require nontrivial integration over the local stress field which are beyond the scope of this paper. Our aim here is to qualitatively compare the trend observed in our experiments to the predicted trend based on an ACEO model, that is the optimal frequency is upscaled as the size parameter is downscaled.

Mathematical derivation for Fig. 5 of the main text

In Fig. 5, our experimental observations are compared to the expected scaling behavior predicted by the model, using $f_{ACEO}^{max}(x) \propto x^{-1}$ based on Eq. S13, employing the half electrode

width $x = w/2$ as the significant scaling parameter for transversal ACEO (see Fig. S1a). Since the geometry differs from the parallel-finger electrodes, the exact values for δR and δC will differ from the solution presented above by some unknown geometrical factor. In order to compensate for that difference, we assume that $\frac{\delta C_{EDL}^{D}(x)}{\delta A(x)}$ is a constant and introduce an empirical parameter $\tilde{\Lambda}$:

$$\tau_C = (2\pi f_{ACEO}^{max}(x))^{-1} = \frac{\delta R * \delta C_{EDL}^{D}}{2} = \tilde{\Lambda} \frac{x}{\lambda_D} \frac{\tau_{DH}}{2} \qquad \text{(S16)}$$

$\tilde{\Lambda}$ is obtained by evaluating Eq. S16 for the lower and upper bounds of the respective frequency range at which the flow pattern was observed for each of the electrodes (see Table S2) and taking the average, yielding

$$\tilde{\Lambda} = 0.6 \pm 0.1$$

Table S2. Data used for the estimation of $\tilde{\Lambda}$

w	$x = w/2$	f	$\lambda_D/(x\pi f \tau_{DH})$
20 µm (lower boundary)	10 µm	2 kHz	1.452
20 µm (upper boundary)	10 µm	10 kHz	0.290
960 nm (lower boundary)	480 nm	80 kHz	0.756
960 nm (upper boundary)	480 nm	320 kHz	0.189
350 nm (lower boundary)	175 nm	200 kHz	0.830
350 nm (upper boundary)	175 nm	3 MHz	0.055
Average (estimate for $\tilde{\Lambda}$)			0.595

With this and plugging Eq. S16 into Eq. S10, the frequency at which the ACEO-inducing stress is maximized at a given x can be obtained:

$$f_{ACEO}^{max}(x) = \frac{\lambda_D}{\tilde{\Lambda}\pi\tau_{DH}} x^{-1}, \qquad \text{(S17)}$$

and consequently, $$\Omega(x, f) = \omega\tau_C \equiv \frac{f}{f_{ACEO}^{max}(x)}$$

$$T_{ACEO}(x, f) \propto \left(\frac{\Omega^2}{(1+\Omega^2)^2}\right) x^{-1}$$

The speed contour lines in Fig. 5 are created by comparing the stress at any given parameter pair (x, f) to the maximum stress at some default parameter x_0 selected within the well-known regime of ACEO[3], the µm and kHz range:

$$\frac{T_{ACEO}(x,f)}{T_{ACEO}^{max}(x_0)} = \frac{4x_0}{x} \frac{\Omega(x,f)^2}{(1+\Omega(x,f)^2)^2} \qquad \text{(S18)}$$

At fixed x, Eq. S18 can be reduced to a quadratic equation in Ω, yielding analytical expressions for the two branches of the iso-stress curves in (x, f) space which are plotted in Fig. 5a:

$$\Omega_{x_0}^{lb}(x) = \frac{1 - \sqrt{1 - x/x_0}}{\sqrt{x/x_0}}$$

$$\Omega_{x_0}^{ub}(x) = \frac{1 + \sqrt{1 - x/x_0}}{\sqrt{x/x_0}}$$

$$\left.\begin{aligned} f_{x_0}^{lb}(x) &= \Omega_{x_0}^{lb}(x) \cdot f_{ACEO}^{max}(x) \\ f_{x_0}^{ub}(x) &= \Omega_{x_0}^{ub}(x) \cdot f_{ACEO}^{max}(x) \end{aligned}\right\} \qquad \text{(S19)}$$

where $f_{x_0}^{lb}, f_{x_0}^{ub}$ are the lower- and upper-limit frequency at which $T_{ACEO}(x, f) \geq T_{ACEO}^{max}(x_0)$, respectively. These functions are plotted in Fig. 5a for x_0 = 100 nm, 1 µm, 10 µm and 100 µm, giving the iso-stress curves which indicate any parameter pairs (x, f) where the ACEO-inducing stress is equal to the calculated maximum for the respective values of x_0, serving as an estimate for the expected magnitude of ACEO flow.

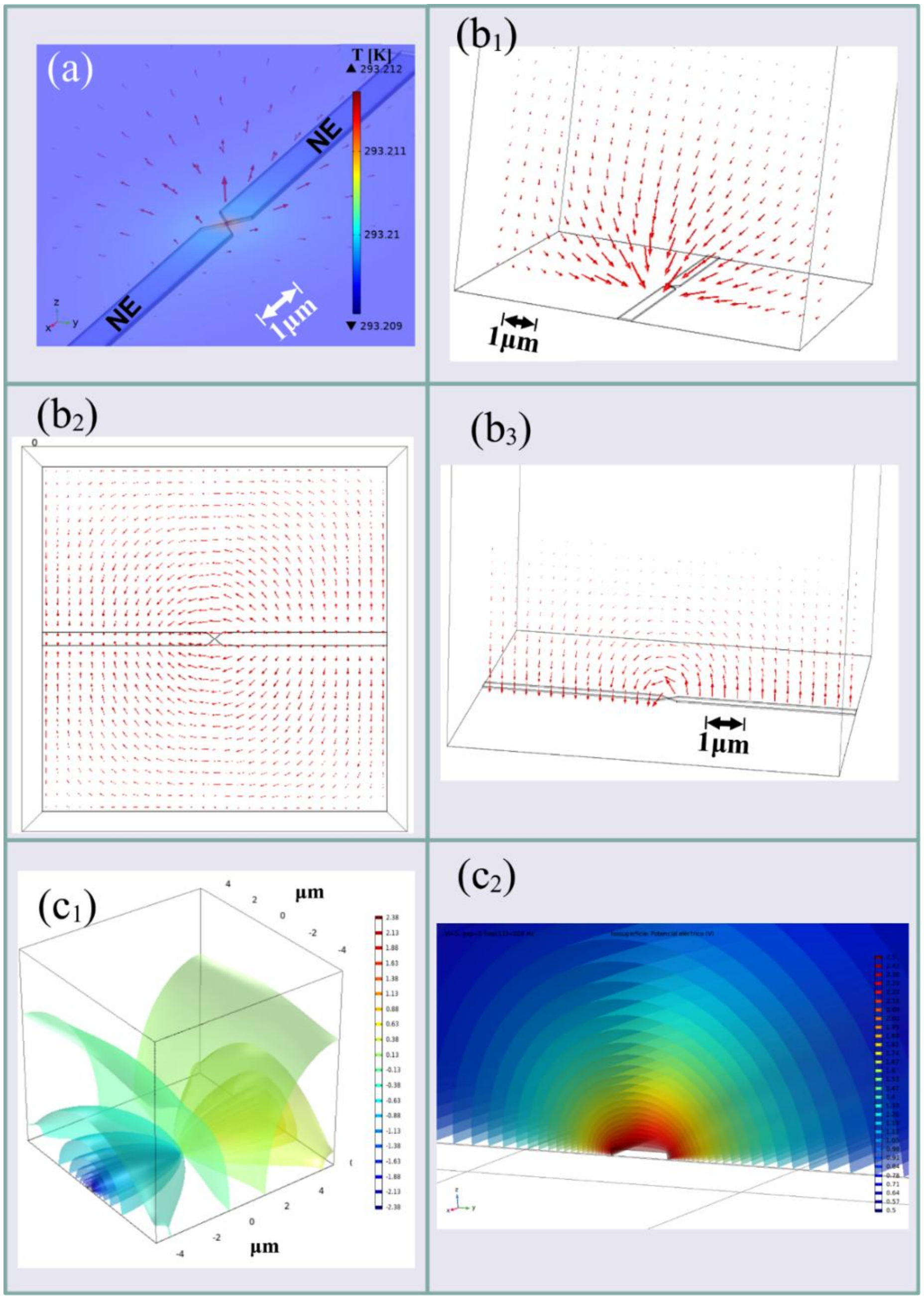

Figure S2. Simulation of Joule heating (a) and various projections of the vector field (b_1-b_3) and iso-potential surfaces (c_1-c_2) of the electric field surrounding the nano-electrode immersed in aqueous solution when voltage is applied. The simulated Joule heating yields a centrosymmetric temperature profile with a maximal temperature increase less than 0.1 K. The electric field has onion-like rotational symmetry. Both the electric field strength and the thermal gradient are highest well within 1μm distance of the electrode nanogap. The context with ETF is explained in detail in the text.

Dielectrophoresis

Positive or negative ***dielectrophoretic (DEP)*** force may influence the flow patterns, as explained in the main text. The supplementary video S8 shows data from a control experiment conducted on a chip of identical design on a $w = 400nm$ electrode with a suspension of $d = 200nm$ fluorescent beads at a comparable buffer conductivity of $22\,\mu S/cm$. The videos show bead accumulation at the nanogap in the range around 320 – 800 kHz, indicating strong DEP force in that range.

While the cross-over frequency of the DEP force[4] does not scale with the electrode size, movement or collection of the same type of PS beads along the electrodes is observed in a much higher frequency range for w = 960 nm electrodes than for the microelectrodes, as shown by the projections in Fig. 4a-b. This trend continues towards electrodes of width $w = 400$ nm, as shown in the supplementary video S8. However, in that experiment the observation of ACEO at frequencies above 800 kHz is hindered by negative DEP force experienced by those beads. The observed particle accumulation above the electrodes is therefore most likely attributed to transversal flow rolls like the ones depicted in Fig. 3, rather than DEP force. Another indicator for the presence of such flow patterns is the sustained longitudinal particle movement along the central axis of the nano electrodes, which would not be possible by DEP alone.

Electrothermal flow

Electrothermal flow (ETF) has been attributed to two distinct causes[5, 6], i.e., heating by absorbed light and Joule heating in the electrode gap. An important factor for the formation of high temperature gradients required for strong ETF is the thermal conductivity of the electrode material. Titanium is known to have a very low thermal conductivity as compared to other metals, thus

suppressing ETF magnitude[7]. Additionally, in contrast to ACEO, ETF magnitude is not expected to scale with electrode size[2], while it is in turn expected to undergo a flow direction transition[8] at a frequency around $f = 3f_{DH}$, due to the interplay of two distinct components, as explained in the ETF theory section below.

Illumination-induced ETF can be neglected for the flow patterns discussed in this work due to the similarity in terms of optical absorptivity of the silicon substrate and titanium electrode material as well as the high heat conductivity of silicon. In order to validate the absence of illumination-induced effect, tests were conducted by changing the intensity and partially blocking the light source. None of these had a noticeable effect on the observed flow patterns.

Joule heating in our system leads to negligible temperature increments, according to our COMSOL simulation, as shown in Fig. S2. However, ETF patterns of limited spatial extent may still be observable, if the temperature gradient and electric field are high locally. In cases where this type of ETF is significant, the simulated heat profile (Fig. S2) suggests the formation of a centrosymmetric flow pattern, which we observed in other instances (see Fig. 4d in the main text) and which is distinct from the pattern we associate with ACEO.

ETF theory

The force causing electrothermal flow $\boldsymbol{F}_{ET}$ is dominated by two terms[8], a Coulomb force $\boldsymbol{F}_{Cou}$ and a dielectric force $\boldsymbol{F}_{Diel}$:

$$F_{ET} = F_{Cou} + F_{Diel}$$

$$F_{Cou} = \frac{A_C}{1+(\omega\tau_{DH})^2}(\boldsymbol{\nabla}T \cdot \boldsymbol{E})\boldsymbol{E} = F_{Cou} * \hat{\boldsymbol{u}}_E$$

$$F_{Diel} = A_D|E|^2\boldsymbol{\nabla}T = F_{Diel} * \hat{\boldsymbol{u}}_{\nabla T} \quad \text{(S20)}$$

with $F_{Cou} \propto (\hat{\boldsymbol{u}}_{\nabla T} \cdot \hat{\boldsymbol{u}}_E) * \nabla T E^2$ and $F_{Diel} \propto \nabla T|E|^2$, where $\omega = 2\pi f$ is the angular frequency of the applied AC electric field $\boldsymbol{E}$, and $\hat{\boldsymbol{u}}_E$and $\hat{\boldsymbol{u}}_{\nabla T}$ are the unit vectors in direction of the local electric

field and temperature gradient, respectively. A_C and A_D are factors that can be considered constant in aqueous solution reasonably close to room temperature[8]. The Coulombic term is suppressed at high frequencies $\omega > \frac{3}{\tau_{DH}}$ and also by means of the vector product, due to the fact that in many places around the gap region, where the electric field is strongest, $\hat{\boldsymbol{u}}_E$ and $\hat{\boldsymbol{u}}_{\nabla T}$ are at nearly perpendicular orientation in respect to one another, as can be seen from the simulation results in Fig. S2. In this geometry one is more likely to observe the dielectric variant of ETF, which acts parallel to $\hat{\boldsymbol{u}}_{\nabla T}$. It can be seen from Fig. S2a that both the direction and magnitude of $\boldsymbol{\nabla}T$ are centrosymmetric around the electrode nanogap, leading us to the conclusion that ETF is much stronger near the electrode gap than on parts of the nano-electrodes far away from the gap region, and flow patterns resembling central symmetry are mostly attributed to ETF.

Supplementary videos

A total of 8 videos listed below are presented corresponding to the simulated ACEO flow in Fig. 3a as well as most of the projections shown in Figs. 3 and 4 of the main text. Video S1 was created by stacking cross-sections obtained from COMSOL together and converting the stack to an mp4 file. The videos S2-S5 were prepared from the original TIFF stacks (with file sizes up to 1GB each) by projecting the maximal intensity of 10 frames, so as to emphasize particle traces, and subsequently converting to mp4 format. In the case of Videos S3, S5 and S8, the original temporal resolution of 100 fps was reduced to 25 fps before mp4 conversion in order to reduce the file size. All of the videos S2-S8 play in real time. Table S3 lists the associated experimental parameters and referring figure to each video.

Video S1: Fly-by animation of simulated ACEO flow (MP4)

Video S2: Flow spirals on microelectrodes (MP4)

Video S3: Flow patterns on w = 960 nm electrodes up to 100 kHz (MP4)

Video S4: Flow patterns on w = 20 μm electrodes (MP4)

Video S5: Flow patterns on w = 960 nm electrodes up to 1 MHz (MP4)

Video S6: Flow patterns on w = 350 nm electrodes (MP4)

Video S7: Flow patterns at 1 MHz on various electrodes (MP4)

Video S8: Flow patterns on w = 400 nm electrodes (MP4)

Table S3. List of supplementary videos

**Video name**	**Figure reference**	**Description**
Video_S1	Fig. $3a_2$	Fly-by animation of simulated ACEO flow cross-sections over electrodes of width w = 400 nm from the electrode gap to a distance of 1.45 μm
Video_S2	Fig. 3d	Flow spirals observed over microelectrodes (w = 20 μm) at an applied field of 2 kHz, 7.5 V_{pp}. 10 frames were projected to facilitate the observation of flow paths.
Video_S3	Fig. 3e-f	Flow patterns observed over w = 960 nm electrodes at varied field frequencies in the range from 2.5 kHz up to 100 kHz around 2 V_{pp}. 10 frames were projected to facilitate the observation of flow paths. In some cases, the observed patterns indicate spiral movement, as exemplified by the snap shots presented in Fig. 3e-f.
Video_S4	Fig. 4a	Flow patterns observed over microelectrodes (w = 20 μm) at varied field frequencies in the range from 2 kHz up to 100 kHz. In comparison to Videos S3 and S5, this clearly demonstrates the frequency shift of the flow patterns in conjunction with size scaling.
Video_S5	Fig. 4b	Flow patterns observed over w = 960 nm electrodes at varied field frequencies in the range from 20 kHz up to 1 MHz. This video complements video S3.
Video_S6	Fig. 4c	Flow patterns observed over w = 350 nm nanoelectrodes at varied field frequencies in the range from 200 kHz up to 3 MHz.
Video_S7	Fig. 4d	Flow patterns observed at an AC frequency of 1 MHz over the electrodes of three different widths (w = 20 μm, w = 960 nm and w = 350 nm) in comparison

Video_S8	none	Flow patterns observed over *w* = 400 nm electrodes at varied field frequencies in the range from 200 kHz up to 1.25 MHz. This video shows DEP immobilization of *d* = 200nm fluorescent beads near the electrode nanogap in the presence of+ ACEO flow at frequencies from 320 to 800 kHz.

References:

1. Ramos, A., et al., *AC Electric-Field-Induced Fluid Flow in Microelectrodes.* Journal of Colloid and Interface Science, 1999. **217**: p. 420-422
2. Castellanos, A., et al., *Electrohydrodynamics and dielectrophoresis in microsystems: scaling laws.* Journal of Physics D: Applied Physics, 2003. **36**(20): p. 2584-2597
3. Squires, T.M. and M.Z. Bazant, *Induced-charge electro-osmosis.* Journal of Fluid Mechanics, 2004. **509**: p. 217-252
4. Green, N.G. and H. Morgan, *Dielectrophoretic separation of nanoparticles.* Journal of Physics D: Applied Physics, 1997. **30**: p. L41-L44
5. Green, N.G., et al., *Electric field induced fluid flow on microelectrodes: the effect of illumination.* Journal of Physics D: Applied Physics, 2000. **33**(2): p. L13-L17
6. Green, N.G., et al., *Electrothermally induced fluid flow on microelectrodes.* Journal of Electrostatics, 2001. **53**(2): p. 71-87
7. Salari, A., et al., *AC Electrothermal Effect in Microfluidics: A Review.* Micromachines (Basel), 2019. **10**(11) 762.
8. Ramos, A., et al., *Ac electrokinetics: a review of forces in microelectrode structures.* Journal of Physics D: Applied Physics, 1998. **31**(18): p. 2338-2353
